# Thermography as a Supporting Tool for the Diagnosis of Patellar Tendon Dysfunctions: A Systematic Review

**DOI:** 10.1055/s-0043-1770971

**Published:** 2024-07-18

**Authors:** Sálvio Tadeu Correia de Barros, Eduardo Borba Neves, Marcos Leal Brioschi

**Affiliations:** 1 Universidade Federal de Alagoas (UFAL), Maceió, AL, Brasil; 2 Programa de Pós-Graduação em Engenharia Biomédica, Universidade Tecnológica Federal do Paraná (PPGEB/UTFPR), Curitiba, PR, Brasil; 3 Pós-Gaduação em Termologia e Termografia Médica, Faculdade de Medicina, Universidade de São Paulo (FMUSP), São Paulo, SP, Brasil

**Keywords:** diagnosis, knee, patellar tendon, thermography

## Abstract

**Objective** The present study aimed to analyze the use of thermal images as a tool to aid in the diagnosis of dysfunctions in the patellar tendon.

**Methods** A systematic review of the literature was carried out, following the PRISMA protocol with a search in international databases: Web of Science, SCOPUS, and Pubmed. Descriptors in English were used with the following combination ("thermal imaging" OR thermography OR "Infrared image" OR "Skin Temperature" OR "thermal image" OR "Infrared imaging") AND ("patellar tendon"). The evaluation of the methodological quality of the selected works was carried out by two evaluators independently, using the Quality Assessment of Diagnostic Accuracy Studies (QUADAS-2) tool.

**Results** Six original articles were selected. Three papers evaluated changes in skin temperature in pathological situations. Two other works evaluated the reliability and reproducibility of thermography specifically in the patellar tendon region, and one article monitored skin temperature before and after two physical training protocols.

**Conclusion** It can be concluded that the use of thermal images proved to be an excellent tool to support the diagnosis of dysfunctions in the patellar tendon region, as well as for monitoring the physiological changes in this region also in non-pathological conditions, such as during physical training.

## Introduction

The knee is the joint most commonly injured by athletes. Millions of adult amateur athletes of both sexes are affected by knee injuries every year. Non-contact knee injuries account for more than two-thirds of knee injuries when data from team sports athletes is analyzed. These injuries result in profound consequences, including physical disability, substantial treatment costs, and even depression in some cases. 1

Lyons et al. 2 analyzed the epidemiological data of injuries of the knee extensor mechanism, using the database of emergency departments in the United States in the period from 2001 to 2020. The authors observed that ruptures of the patellar tendon are the second most frequent type of injury (13.5% of all injuries, overall incidence rate: 0.48), second only to patellar fractures (77.5% of all injuries, overall incidence rate: 2.69).

Patellar tendon dysfunction is usually the result of a chronic process or an acute trauma. Chronic inflammation, such as patellar tendonitis, weakens the tendon and increases the likelihood of rupture, either partial or complete. In addition, some systemic pathologies (systemic lupus erythematosus, chronic kidney disease, and diabetes mellitus, among others) can lead to a weakened tendon that is predisposed to rupture. 3

Patellar tendinopathy is a degenerative condition marked by histopathological changes in the tendon. Patellar tendinopathy, also commonly called jumper's knee, most commonly occurs in active individuals between the ages of 14 and 40. 4 The clinical diagnosis of patellar tendinopathy is based on the patient's symptoms, and can be characterized by pain on palpation at the lower pole of the patella and adjacent areas and, in more advanced cases, a palpable nodule and associated edema may be present. 5 Complementary exams, such as radiography, ultrasonography (US) and magnetic resonance imaging (MRI) help the diagnosis, as they can define the exact location of the lesion, its extent, as well as allow the visualization of degenerative changes, when present. 5

Magnetic resonance imaging is the method with the best resolution to support the diagnosis of patellar tendon injury, as it allows observation and evaluation of bone structures and soft tissues. 3 Recently, the use of thermal imaging in the evaluation and follow-up of patients with musculoskeletal injuries has shown great relevance in clinical practice. 6 7 8 Medical thermography allows a non-invasive, low-cost and non-radioactive analysis, 9 resulting in a functional and visual measurement of skin temperature distribution. In this sense, the present work aimed to analyze the use of thermal images as a tool to aid in the diagnosis of dysfunctions in the patellar tendon.

## Methodology

**Fig. 1:**
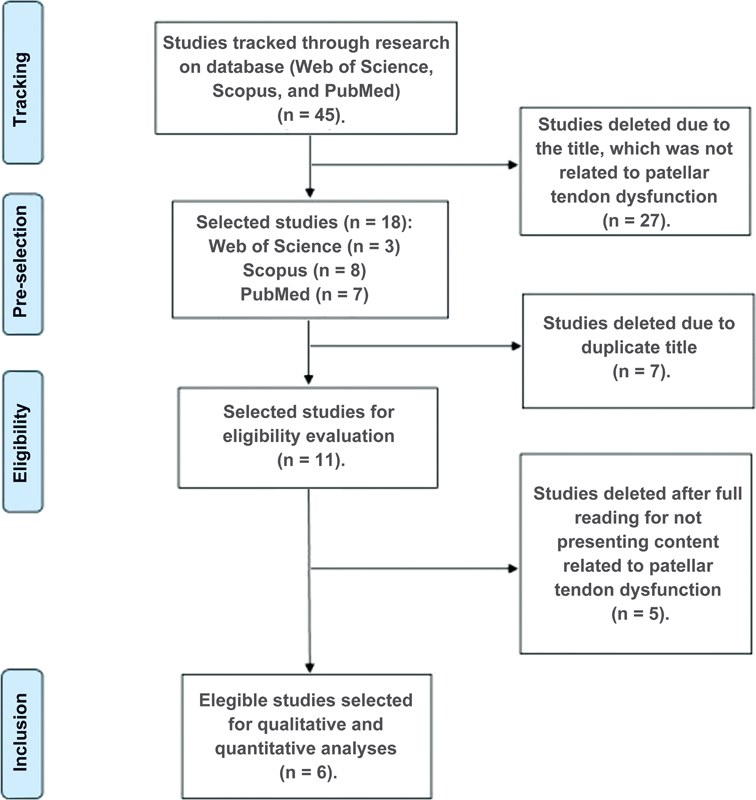
Flowchart of the search and selection process of articles included in this systematic review.

A literature review was carried out, which followed the PRISMA protocol for systematic review studies. Therefore, we used a search in 3 of the largest international databases (Web of Science, SCOPUS, and Pubmed), according to the keywords and database presented in Table 1 , and the selection flow presented in Fig. 1 .

**Table 1:** Presentation of databases and search phrases used in this systematic review

Databases	Search phrases
**Web of Science**	All fields ( "patellar tendon" ) AND All fields ( "thermal imaging" OR thermography OR "Infrared image" OR "Skin Temperature" OR "thermal image" OR "Infrared imaging" )
**Scopus**	TITLE-ABS-KEY ( "patellar tendon" ) AND TITLE-ABS-KEY ( "thermal imaging" OR thermography OR "Infrared image" OR "Skin Temperature" OR "thermal image" OR "Infrared imaging" )
**PubMed**	("thermal imaging" OR thermography OR "Infrared image" OR "Skin Temperature" OR "thermal image" OR "Infrared imaging") AND ("patellar tendon")

After the initial search, using the search phrases mentioned in Table 1 , two independent evaluators carried out the selection of studies. In cases of disagreement between the evaluators, a third researcher gave his opinion.

This systematic review included studies that assessed skin temperature in dysfunctions in the patellar tendon region. Studies from systematic reviews, reviews with meta-analysis, case studies, and studies without full text were excluded. Studies with a publication date prior to 1985 were also excluded, in view of the low resolution of older camera images.

The following data were extracted from the studies: sample profile, type of intervention, main results, and delta temperature observed by infrared thermography.

The evaluation of the methodological quality of the selected works was carried out by two evaluators independently using the Quality Assessment of Diagnostic Accuracy Studies instrument 10 ( Table 2 - Quadas-2). 11 12 13 14 15 16

**Table 2:** Assessment of methodological quality and risk of bias of studies selected by the Quality Assessment of Diagnostic Accuracy Studies (QUADAS-2) tool

	Risk of bias				Applicability concerns		
Study	Selection of participants	Index test	Reference standard	Flow and timing	Patient selection	Index test	Reference standard
Molina-Payá et al. 2021 11	+	+	+	+	+	+	+
Dos Anjos et al. 12	+	+	+	+	+	+	+
Alfieri, 2020 13	+	+	+	+	+	+	+
Sanz-López et al., 2016 14	+	+	+	+	+	+	+
Gross et al. 1989 15	+	+	−	+	+	+	−
Mangine et al. 1987 16	+	+	+	+	+	+	+

+: High risk; -: Low risk.

## Results

Six original articles were selected to compose the corpus of this review. Three papers evaluated changes in skin temperature in pathological situations. Two other works evaluated the reliability and reproducibility of thermography specifically in the patellar tendon region, and one article monitored skin temperature before and after two physical training protocols. The flowchart illustrating the article selection process is shown in Fig. 1 . Table 3 presents the data of interest extracted from each of the analyzed studies. 11 12 13 14 15 16

**Table 3:** Distribution of selected articles according to author (year), sample, intervention, delta temperature and results found

Author (year)	Sample	Intervention	Difference observed in skin temperature	Results
Molina-Payá et al. 2021 11	17 participants (7 women and 10 men) aged between 18 and 62 years (mean: 32.2 years; SD: 10.9 years)	Static position for assessing intra- and inter-examiner reliability and reproducibility	Intra-examiner differences were 0.006°C (Limits of Agreement: -0.10 to 0.10), and inter-examiner differences were 0.001°C (Limits of Agreement: -0.13 to 0.13).	Skin temperature was similar at intra-examiner (F1.33 = 0.488; p = 0.490) and inter-examiner (F1.33 = 0.011; p = 0.917).
Dos Anjos et al. 12	25 subjects with bilateral osteoarthritis (OA), aged 54 to 78 years.	Comparisons between symptomatic and asymptomatic knees did not reveal differences according to Kellgren-Lawrence classification, knee surface temperature or Pressure Pain Threshold (PPT)	Symptomatic knees = 31.2 ± 1.4; asymptomatic knees = 31.1 ± 1.5, (p value for paired t test = 0.379)	Patients with bilateral knee OA showed no difference in symptomatic and asymptomatic knees in relation to Kellgren-Lawrence rating scale, knee temperature, and pain tolerance.
Alfieri, 2020 13	11 volunteers of both genders (63.1 ± 9.5 years) participated in this study. All with unilateral osteoarthritis of the knee	Voluntary thermographic evaluation by an infrared sensor (FLIR T650SC). The anterior region of the thigh, leg and knee were evaluated. Pain pressure threshold (PPT) were evaluated by algometry in the vastus medialis, vastus lateralis, rectus femoris and patellar tendon.	When comparing skin temperature, only the knee region showed a significant difference between the sides (affected knees = 31.2 ± 1.4; unaffected knees = 29.2 ± 1.2, (p value for paired t test = 0.03)	Individuals with knee osteoarthritis had higher temperature in the affected knee when compared to the contralateral one, but this was not associated with pain pressure thresholds.
Sanz-López et al., 2016 14	20 male volunteers, aged between 18 and 25 years were divided into 2 groups with 10 subjects each.	A group trained with eccentric overload and 3 sessions of continuous running, and another group trained only with 3 sessions of continuous running. Infrared thermography (IRT) was used to assess skin temperature over the patellar tendon before, shortly after, and after 10 minutes of running.	Skin temperature measurements, in the region of the patellar tendon, taken 10 minutes after the running intervention in the group trained with eccentric overload showed statistically higher values than those obtained before running in the first and third (differences = 0.81 °C and 1. 91 °C, p <0.05, respectively)	The thermographic images of the patellar tendon did not show any statistically different behavior between the groups. Although significant differences were observed between the evaluation times, within each group.
Gross et al., 1989 15	17 volunteers, six men and eleven women, aged between 19 and 65 years.	The study had two purposes. The first was to evaluate the reliability of a method for quantitative assessment of thermograms. The second objective was to test the hypotheses of difference in skin temperature over the patellar tendon between dominant and non-dominant limbs.	Intra-rater reliability was r = 0.99. Inter-rater reliability (two investigators) was r = 0.88. The average temperature measured on the patellar tendon for the dominant and non-dominant limbs did not show statistically significant differences. (dominant = 29.61 ± 0.52; non-dominant = 29.60 ± 0.96).	The presented thermogram analysis method can be used reliably. There is no change in the thermal pattern of the patellar tendon as a function of limb dominance.
Mangine, 1987 16	17 patients diagnosed with patellar tendinitis, seven men and ten women aged between 12 and 28 years.	The intent of this study was to determine whether the thermal pattern of tendinitis can be distinguished using infrared thermography. Infrared heat emissions were recorded with a resolution of 0.05°C. With legs open and elevated, subjects were thermalized for a period of 10 minutes at the temperature of the image acquisition room. The ambient temperature was maintained free from drafts at 20.0 ± 1.0°C.	In volunteers who had unilateral dysfunction, the mean difference in skin temperature over the patellar tendon between the two sides was 1.23 °C.	An identifiable thermal pattern associated with patellar tendonitis was found in most of the evaluated subjects (78%). Thermography seems useful as a non-invasive and objective method of detecting soft tissue inflammation around the patellar tendon, and also helps to differentiate this disease from other pathologies of the knee.

## Discussion

This study aimed to analyze the use of thermal images as a tool to aid in the diagnosis of dysfunctions in the patellar tendon. Therefore, we started the discussion of the results by analyzing the reliability and reproducibility of thermography in the region of the patellar tendon.

Gross et al. 15 and Molina-Payá et al. 11 reported excellent reliability and reproducibility values. Molina-Payá et al. 11 reported ICC values between 0.904 and 0.998. These values correspond to a difference in temperature of the order of 0.006°C (Limits of Agreement: -0.10 to 0.10) intra-examiner, and 0.001°C (Limits of Agreement: -0.13 to 0.13) inter-examiner. In the study by Gross et al. 15 the Pearson correlation coefficient was used to assess reliability, reporting intra-examiner reliability values of 0.99 and inter-examiner (two investigators) of 0.88. Inter-examiner reliability was also investigated in the study by Selfe et al. 17 involving patients with pain in the anterior region of the knee, indicating good results. They observed ICC values between 0.82 and 0.97 when using thermally inert markers, placed in specific anatomical locations, defining an area on the anterior aspect of the knee.

The reliability values and reproducibility of thermography in the patellar tendon region are in line with what is observed in other body regions, allowing its safe use in clinical practice. Dos Santos Moraes et al. 18 aimed to evaluate the inter- and intra-examiner reliability of infrared thermography in the analysis of skin temperature in people complaining of pain in the upper trapezius muscle. Eighty-two individuals of both sexes who had moderate or severe pain in the upper trapezius muscle were evaluated. Area evaluation indicated a very strong overall intraclass correlation coefficient (ICC) for both intra-examiner (between 0.936 and 0.979) and inter-examiner (between 0.933 and 0.979).

Dos Anjos et al., 12 Alfieri 13 and Mangine et al. 16 reported significant variations in skin temperature ( Table 3 ) over the patellar tendon region in conditions of pathophysiological changes diagnosed by gold standard methods. The studies analyzed in this review suggest that thermography is capable of documenting thermal variations at the level of the skin, resulting from pathophysiological changes in the region of the patellar tendon. 12 13 16

This ability to capture thermal changes resulting from alterations in human physiology has allowed the use of thermal images in several medical applications, such as: diagnosis of musculoskeletal injuries, 8 monitoring changes in the feet of diabetics, 19 aiding in the diagnosis of Osgood-schlatter disease 20 , and others. In this sense, it can be said that thermography has been consolidated in medical practice as a reliable tool with a clinical application that permeates several specialties.

In addition to the applications more directed to the medical area, Sanz-López et al. 14 involved the practice of physical training and the monitoring of the thermal response at the level of the patellar tendon. The authors observed significant changes in skin temperature over the patellar tendon after continuous running training and eccentric strength training associated with continuous running training. Bandeira et al. 6 and Neves et al. 21 had already reported similar findings in other body regions. These observations corroborate the applicability of using thermal images to assess the patellar tendon region even in non-pathological situations, such as monitoring physical training, 6 21 whether for injury prevention or even for workload assessment.

As limitations of the present review, the low number of studies that used thermal imaging of the patellar tendon region available in the literature, and the heterogeneity of the patients of the analyzed studies, variables and interventions used can be mentioned. However, on the other hand, this aforementioned heterogeneity allowed a holistic approach to the applicability of the tool to the evaluation of the patellar tendon.

## Conclusion

After analyzing the corpus of works selected in this systematic literature review, it can be concluded that the use of thermal images proved to be an excellent tool to support the diagnosis of dysfunctions in the patellar tendon region, as well as for monitoring the physiological changes in this region also under non-pathological conditions, such as during physical training.

The use of thermography in the clinical practice of health professionals who need to monitor and/or evaluate the patellar tendon is recommended, as well as the development of more original studies, which address other pathologies and non-pathological situations, in order to expand the knowledge of the thermal physiology of this specific region.
