# Supplementary material for: Thermography as a Supporting Tool for the Diagnosis of Patellar Tendon Dysfunctions: A Systematic Review
Source: Rev Bras Ortop (Sao Paulo). 2024 Jul 18;59(5):e657–62. doi: 10.1055/s-0043-1770971 (PMC11624944; doi:10.1055/s-0043-1770971)
Supplement: Related texts

# Research article: A termografia no apoio ao diagnóstico de disfunções no tendão patelar: Uma revisão sistemática

**Keywords:** diagnóstico, joelho, tendão patelar, termografia

## Abstract

**Objetivo** O presente trabalho teve como objetivo analisar o uso das imagens térmicas como ferramenta de auxílio ao diagnóstico de disfunções no tendão patelar.

**Métodos** Realizou-se uma revisão sistemática da literatura, seguindo o protocolo PRISMA com busca nas bases de dados internacionais: *Web of Science, SCOPUS, e Pubmed* ***.*** Foram utilizados descritores em Inglês com a seguinte combinação *("thermal imaging" OR thermography OR "Infrared image" OR "Skin Temperature" OR "thermal image" OR "Infrared imaging") AND ("patellar tendon").* A avaliação da qualidade metodológica dos trabalhos selecionados foi realizada por dois avaliadores de forma independente pelo instrumento *Quality Assessment of Diagnostic Accuracy Studies* (QUADAS-2).

**Resultados** Foram selecionados seis artigos originais. Três trabalhos avaliaram as alterações da temperatura da pele em situações patológicas. Outros dois trabalhos avaliaram a confiabilidade e reprodutibilidade da termografia especificamente na região do tendão patelar, e um artigo monitorou a temperatura da pele antes e após dois protocolos de treinamento físico.

**Conclusão** Pode-se concluir que o uso das imagens térmicas se mostrou uma excelente ferramenta de apoio ao diagnóstico a disfunções na região do tendão patelar, bem como para o monitoramento das alterações fisiológicas dessa região também em condições não patológicas, como durante a prática de treinamento físico.

## Introdução

O joelho é a articulação mais comumente lesionada por praticantes de esportes. Milhões de atletas amadores adultos, de ambos os sexos são acometidos por lesões nos joelhos todos os anos. As lesões sem contato no joelho são responsáveis por mais de dois terços dos traumas de joelho quando analisados os dados de atletas de esportes coletivos. Essas lesões resultam em consequências profundas, incluindo incapacidade física, custos substanciais com o tratamento, e até depressão, em alguns casos. 1

Lyons et al. 2 analisou os dados epidemiológicos das lesões do mecanismo extensor do joelho, por meio do banco de dados dos departamentos de emergência dos Estados Unidos no período de 2001 a 2020. Os autores observaram que as rupturas do tendão patelar são o segundo tipo lesão mais frequente (13,5% de todas as lesões, taxa de incidência geral: 0,48), ficando atrás somente das fraturas da patela (77,5% de todas as lesões, taxa de incidência geral: 2,69).

As disfunções do tendão patelar geralmente é o resultado de um processo crônico ou de um trauma agudo. A inflamação crônica, como a tendinite patelar, enfraquece o tendão e aumenta a probabilidade de ruptura, seja parcial ou completa. Além disso, algumas patologias sistemicas (lúpus eritematoso sistêmico, doença renal crônica, e diabetes mellitus, entre outras) podem levar a um tendão enfraquecido e predisposto à ruptura. 3

A tendinopatia patelar é uma condição degenerativa marcada por alterações histopatológicas no tendão. A tendinopatia patelar, também comumente chamada de joelho do saltador, ocorre mais comumente em indivíduos ativos na faixa etária entre 14 e 40 anos. 4 O diagnóstico clínico de tendinopatia do patelar é baseado nos sintomas do paciente, e pode ser caracterizado por dor à palpação no polo inferior da patela e adjacências e, nos casos mais avançados, nódulo palpável e edema associado podem ser visualizados presentes. 5 Exames complementares, como radiografia, ultrassonografia (US) e ressonância magnética (RM) auxiliam o diagnóstico, pois podem definir o local exato da lesão, sua extensão, bem como permitir a visualização de alterações degenerativas, quando presentes. 5

A ressonância magnética é o método com melhor resolução para o apoio ao diagnóstico de lesão no tendão patelar, pois permite a observação e avaliação das estruturas ósseas e tecidos moles. 3 Recentemente, o uso de imagens térmicas na avaliação e acompanhamento de pacientes com lesões musculoesqueléticas tem apresentados grande relevância na prática clínica. 6 7 8 A termografia médica, permite uma análise não invasiva, de baixo custo e não radioativa, 9 apresentando como resultado uma medida funcional e visual da distribuição da temperatura da pele. Neste sentido, o presente trabalho teve como objetivo analisar o uso das imagens térmicas como ferramenta de auxílio ao diagnóstico de disfunções no tendão patelar.

## Metodologia

Realizou-se de uma pesquisa de revisão da literatura, que seguiu o protocolo PRISMA para estudos de revisão sistemática. Sendo assim, utilizou-se de uma busca em 3 das maiores bases de dados internacionais ( ***Web of Science, SCOPUS, e Pubmed*** ) , conforme as palavras chaves e base de dados apresentadas na Tabela 1 , e o fluxo de seleção apresentado na Fig. 1 .

**Fig. 1:** Fluxograma do processo de busca e seleção dos artigos incluídos na presente revisão sistemática.

**Tabela 1:** Apresentação das bases de dados e frases de busca utilizadas na presente revisão sistemática

Base de dados	Frase de busca
**Web of Science**	Todos os campos ( "patellar tendon" ) AND Todos os campos ( "thermal imaging" OR thermography OR "Infrared image" OR "Skin Temperature" OR "thermal image" OR "Infrared imaging" )
**Scopus**	TITLE-ABS-KEY ( "patellar tendon" ) AND TITLE-ABS-KEY ( "thermal imaging" OR thermography OR "Infrared image" OR "Skin Temperature" OR "thermal image" OR "Infrared imaging" )
**PubMed**	("thermal imaging" OR thermography OR "Infrared image" OR "Skin Temperature" OR "thermal image" OR "Infrared imaging") AND ("patellar tendon")

Após a busca inicial, utilizando as frases de busca citadas na Tabela 1 , dois avaliadores independentes realizaram a seleção dos estudos. Nos casos de discordância entre os avaliadores, um terceiro pesquisador pronunciava seu parecer.

Foram incluídos nesta revisão sistemática estudos que avaliaram a temperatura da pele em disfunções na região do tendão patelar. estudos de revisões sistemáticas, revisões com metanálise, estudos de caso, e estudos sem texto completo foram excluídos. Também foram excluídos estudos com data de publicação anterior ao ano 1985, tendo em vista a baixa resolução das imagens de câmeras mais antigas.

Foram extraídos dos estudos os seguintes dados: perfil da amostra, tipo de intervenção, principais resultados, e delta de temperatura observado pela termografia infravermelha.

A avaliação da qualidade metodológica dos trabalhos selecionados foi realizada por dois avaliadores de forma independente pelo instrumento *Quality Assessment of Diagnostic Accuracy Studies* 10 ( Tabela 2 - Quadas-2). 11 12 13 14 15 16

**Tabela 2:** Avaliação da qualidade metodológica e risco de viés dos estudos selecionados pelo instrumento *Quality Assessment of Diagnostic Accuracy Studies* (QUADAS-2)

	Risco de viés				Preocupações com a Aplicabilidade		
Estudo	Seleção de participantes	Teste índice	Padrão de referência	Fluxo e tempo	Seleção de participantes	Teste índice	Padrão de referência
Molina-Payá et al. 2021 11	+	+	+	+	+	+	+
Dos Anjos et al. 12	+	+	+	+	+	+	+
Alfieri, 2020 13	+	+	+	+	+	+	+
Sanz-López et al., 2016 14	+	+	+	+	+	+	+
Gross et al. 1989 15	+	+	−	+	+	+	−
Mangine et al. 1987 16	+	+	+	+	+	+	+

+: Baixo risco; -: Alto risco.

## Resultados

Foram selecionados 6 artigos originais para compor o corpus desta revisão. Três trabalhos avaliaram as alterações da temperatura da pele em situações patológicas. Outros dois trabalhos avaliaram a confiabilidade e reprodutibilidade da termografia especificamente na região do tendão patelar, e um artigo monitorou a temperatura da pele antes e após dois protocolos de treinamento físico. O fluxograma de ilustração do processo de seleção dos artigos está apresentado na Fig. 1 . A Tabela 3 apresenta os dados de interesse extraído de cada um dos estudos analisados. 11 12 13 14 15 16

**Tabela 3:** Distribuição dos artigos selecionados conforme autor (ano), amostra, intervenção, delta de temperatura e resultados encontrados

Autor (ano)	Amostra	Intervenção	Diferença observada na Temperatura da pele	Resultados
Molina-Payá et al. 2021 11	17 participantes (7 mulheres e 10 homens) entre 18 e 62 anos (média: 32,2 anos; DP: 10,9 anos)	Posição estática para avaliação da confiabilidade e reprodutibilidade intra e inter-avaliador	Intra-examinador foi de 0,006°C (Limites de Concordância: -0,10 a 0,10), e as diferenças inter-examinadores foram de 0,001°C (Limites de Concordância: -0,13 a 0,13).	a temperatura da pele foi semelhante à intra-examinador (F1,33 = 0,488; p = 0,490) e inter-examinador (F1,33 = 0,011; p = 0,917).
Dos Anjos et al. 12	25 indivíduos com osteoartrite (OA) bilateral, com idades variando de 54 a 78 anos.	Comparações entre joelhos sintomáticos e assintomáticos não revelaram diferenças de acordo com a classificação de Kellgren-Lawrence, temperatura superficial do joelho ou Limiar de Dor à Pressão (LDP)	Joelhos sintomáticos = 31.2 ± 1.4; joelhos assintomáticos = 31.1 ± 1.5, (p valor para o Teste t pareado = 0.379)	Pacientes com OA bilateral de joelho não apresentaram diferenças em joelhos sintomáticos e assintomáticos em relação escala de classificação de Kellgren-Lawrence, temperatura do joelho, e tolerância à dor (LDP).
Alfieri, 2020 13	11 voluntários de ambos os sexos (63,1 ± 9,5 anos) participaram deste estudo. Todos com osteoartrite unilateral do joelho	Avaliação termográfica voluntária por um sensor infravermelho (FLIR T650SC). foram avaliados a região anterior da coxa, perna e o joelho. Os limiares de dor à pressão (LPP) foram avaliados por algometria no vasto medial, vasto lateral, reto femoral e tendão patelar.	Ao comparar a temperatura da pele, apenas a região do joelho apresentou diferença significativa entre os lados (joelhos acometido = 31.2 ± 1.4; joelhos não acometido = 29.2 ± 1.2, (p valor para o Teste t pareado = 0.03)	Indivíduos com osteoartrite de joelho apresentaram maior temperatura do joelho acometido quando comparado com o contralateral, mas isso não foi associado aos limiares de dor à pressão.
Sanz-López et al., 2016 14	20 voluntários do sexo masculino, com faixa etária entre 18 e 25 anos foram divididos em 2 grupos com 10 sujeitos cada.	Um grupo treinado com sobrecarga excêntrica e 3 sessões de corrida contínua, e outro grupo treinado apenas com 3 sessões de corrida contínua. Foi utilizada a termografia infravermelha (IRT) para avaliação da temperatura da pele sobre o tendão patelar antes, logo depois, e depois de 10 minutos da corrida.	As medidas da temperatura da pele, na região do tendão patelar, realizadas 10 minutos após a intervenção de corrida no grupo treinado com sobrecarga excêntrica mostraram valores estatisticamente superiores aos obtidos antes da corrida no primeiro e terceiro (diferenças = 0,81 °C e 1,91 °C, p <0,05, respectivamente)	As imagens termográficas do tendão patelar não mostraram qualquer comportamento estatisticamente diferente entre os grupos. Embora tenha-se observado diferenças significativas entre os momentos de avaliação, dentro de cada grupo.
Gross et al., 1989 15	17 voluntários, seis homens e onze mulheres, com idades entre 19 e 65 anos.	O estudo teve dois propósitos. O primeiro foi avaliar a confiabilidade de um método para avaliação quantitativa de termogramas. O segundo objetivo foi testar a hipóteses de diferença na temperatura da pele sobre o tendão patelar entre os membros dominantes e não dominantes.	Confiabilidade intra-avaliador foi r = 0,99. Confiabilidade entre avaliadores (dois investigadores) foi r = 0,88. A média das temperaturas medidas sobre o tendão patelar para os membros dominante e não dominante não apresentaram diferenças estatisticamente significativas. (dominante = 29.61 ± 0.52; não dominante = 29.60 ± 0.96).	O método de análise de termogramas apresentado pode ser usado de forma confiável. Não há alteração do padrão térmico do tendão patelar em função da dominância do membro.
Mangine, 1987 16	17 pacientes diagnosticados com tendinite patelar, sete homens e dez mulheres com idades entre 12 e 28 anos.	A intenção deste estudo foi determinar se o padrão térmico da tendinite pode ser distinguido usando termografia infravermelha. As emissões de calor infravermelho foram registradas com resolução de 0,05°C. Com as pernas abertas e elevadas, os sujeitos termalizados por um período de 10 minutos com a temperatura da sala de aquisição das imagens. A temperatura ambiente foi mantido livre de correntes de ar em 20,0 ± 1,0°C.	Nos voluntários que apresentavam disfunção unilateral, a diferença média da temperatura da pele sobre o tendão patelar entre os dois lados foi de 1,23 °C.	Um padrão térmico identificável associado a tendinite patelar foi encontrada na maioria dos sujeitos avaliados (78%). A termografia parece útil como um método não invasivo e objetivo de detectar inflamação dos tecidos moles ao redor do tendão patelar, e também ajuda a diferenciar esta doença de outras patologias do joelho.

## Discussão

O presente trabalho teve por objetivo analisar o uso das imagens térmicas como ferramenta de auxílio ao diagnóstico de disfunções no tendão patelar. Para tanto, iniciamos a discussão dos resultados analisando a confiabilidade e a reprodutibilidade da termografia na região do tendão patelar.

Gross et al. 15 e Molina-Payá et al. 11 reportaram excelentes valores de confiabilidade e a reprodutibilidade. Molina-Payá et al. 11 reportaram valores de ICC entre 0,904 e 0,998. Esses valores correspondem a uma diferença de temperatura da ordem de 0,006°C (Limites de Concordância: -0,10 a 0,10) intra-examinador, e 0,001°C (Limites de Concordância: -0,13 a 0,13) inter-examinadores. Já no estudo de Gross et al. 15 usou o coeficiente de correlação Pearson para avaliar a confiabilidade, reportando valores de confiabilidade intra-avaliador de 0,99 e inter-avaliadores (dois investigadores) de 0,88. A confiabilidade inter-examinador também foi investigada no estudo de Selfe et al. 17 envolvendo pacientes com dor na região anterior no joelho, indicando bons resultados. observaram valores de ICC entre 0,82 e 0,97 ao utilizar marcadores termicamente inertes, colocados em locais anatômicos específicos, definindo uma área sobre a face anterior do joelho.

Os valores de confiabilidade e a reprodutibilidade da termografia na região do tendão patelar estão em linha com o que se observa em outras regiões corporais, permitindo o seu uso com segurança na prática clínica. Dos Santos Moraes et al. 18 objetivou avaliar a confiabilidade inter e intra-examinadores da termografia infravermelha na análise da temperatura da pele de pessoas com queixa de dor no músculo trapézio superior. Foram avaliados 82 indivíduos de ambos os sexos que apresentavam dor moderada ou intensa no músculo trapézio superior. A avaliação das áreas indicou um coeficiente de correlação intraclasse (ICC) geral muito forte para ambos intra examinador (entre 0,936 e 0,979) e inter-examinador (entre 0,933 e 0,979).

Dos Anjos et al., 12 Alfieri 13 e Mangine et al. 16 reportaram variações significativas na temperatura da pele ( Tabela 3 ) sobre a região do tendão patelar em condições de alterações fisiopatológicas diagnosticadas por métodos padrão-ouro. Os estudos analisados nesta revisão sugerem que a termografia é capaz de documentar as variações térmicas ao nível da pele, decorrentes das alterações fisiopatológicas na região do tendão patelar. 12 13 16

Essa capacidade de captar as alterações térmicas decorrentes das alterações na fisiologia humana tem permitido o uso das imagens térmicas em diversas aplicações médicas, tais como: diagnóstico de lesões musculoesqueléticas, 8 acompanhamento de alterações nos pés de diabéticos, 19 auxílio ao diagnóstico da doença de Osgood-schlatter, 20 entre outras. Neste sentido, pode-se dizer que a termografia tem se consolidado na prática médica como uma ferramenta confiável e com uma aplicação clínica que perpassa diversas especialidades.

Além das aplicações mais direcionadas à área médica, Sanz-López et al. 14 envolveu a prática de treinamento físico e o acompanhamento da resposta térmica ao nível do tendão patelar. Os autores observaram modificações significativas na temperatura da pele sobre o tendão patelar após a realização de treinamentos de corridas contínuas e treinamento de força excêntrico associado ao treino de corrida contínua. Bandeira et al. 6 e Neves et al. 21 já haviam reportado achados semelhantes em outras regiões corporais. Essas observações corroboram a aplicabilidade do uso das imagens térmicas para avaliação da região do tendão patelar mesmo em situações não patológicas, como no acompanhamento do treinamento físico, 6 21 seja para prevenção de lesões, ou mesmo para avaliação da carga de trabalho.

Pode-se citar como limitações da presente revisão o baixo número de estudos que usaram o imageamento térmico da região do tendão patelar disponível na literatura, e a heterogeneidade dos objetivos dos estudos analisados, variáveis e intervenções utilizadas. Contudo, por outro lado, essa heterogeneidade supracitada permitiu uma abordagem holística sobre a aplicabilidade da ferramenta à avaliação do tendão patelar.

## Conclusão

Após a análise do corpus de trabalhos selecionados nesta revisão sistemática da literatura, pode-se concluir que o uso das imagens térmicas se mostrou uma excelente ferramenta de apoio ao diagnóstico a disfunções na região do tendão patelar, bem como para o monitoramento das alterações fisiológicas dessa região também em condições não patológicas, como durante a prática de treinamento físico.

Recomenda-se o uso da termografia na prática clínica dos profissionais de saúde que tenham necessidade de monitorar e/ou avaliar o tendão patelar, bem como o desenvolvimento de mais estudos originais, que abordem outras patologias e situações não patológicas, para que se amplie o conhecimento da fisiologia térmica dessa região em específico.
